# Study of Protoscolicidal Effects of Hypertonic Glucose on Protoscolices of Hydatid Cyst at Different Concentrations and Exposure Times

**DOI:** 10.1155/2014/314502

**Published:** 2014-10-29

**Authors:** Mojtaba Shahnazi, Fatemeh Badakhsh, Abbas Azadmehr, Mehrzad Saraei, Mahmood Alipour, Maryam Shahnazi, Mehri Jamshidi

**Affiliations:** ^1^Department of Parasitology, Qazvin University of Medical Sciences, Qazvin, Iran; ^2^Cellular & Molecular Research Institute, Qazvin University of Medical Sciences, Qazvin, Iran; ^3^Department of Immunology, Qazvin University of Medical Sciences, Qazvin, Iran; ^4^Department of Social Medicine, Qazvin University of Medical Sciences, Qazvin, Iran; ^5^Zanjan University of Medical Sciences, Zanjan, Iran; ^6^Department of Pediatrics, Ghods Hospital, Qazvin University of Medical Sciences, Qazvin, Iran

## Abstract

Surgical operation is the treatment of choice for hydatid cyst. To date, many protoscolicidal substances have been used for inactivation of hydatid cyst contents but most of these compounds may produce several side effects. The aim of this study was to evaluate the protoscolicidal effects of hypertonic glucose on protoscolices of hydatid cyst at different concentrations and exposure times. Protoscolices were obtained aseptically from the livers of slaughtered sheep at Qazvin abattoir, Iran. Protoscolices were exposed to different concentrations of hypertonic glucose (10%, 15%, 20%, 25%, 30%, 40%, and 50%) at different times (1, 2, 3, 4, 5, 6, 7, 8, 9, 10, 20, 30, 40, 50, and 60 min). Viability of protoscolices was evaluated by 0.1% eosin and the movement of protoscolices flame cells. The highest protoscolicidal effect (100%) of hypertonic glucose was obtained at concentrations 40% and 50% following 40 and 20 min exposure times, respectively. Some protoscolicidal agents show a variety of dangerous complications such as biliary tract fibrosis and liver necrosis; however, hypertonic glucose especially at a concentration of 40% may demonstrate less side effects compared with glucose 50%. Further in vivo investigations are suggested.

## 1. Introduction

Hydatidosis is one of the most important zoonotic diseases with worldwide distribution. Although hydatid disease is mostly found in liver and lung, it can arise anywhere in the body [1]. Currently, there are three options for the treatment of liver hydatid disease, where, among them, surgical operation is considered as the most efficient treatment. [2, 3]. Spillage of the cyst contents is very common, despite taking technical precautions. This is the major cause of recurrence, which is seen in approximately 10% of postoperative cases. During the operation and discharging of the cyst, the injection of protoscolicidal agents into hydatid cyst is performed to prevent the spread of infection and secondary cyst formations [2, 4, 5]. To date, many protoscolicidal substances (formalin, hydrogen peroxide, cetrimide, pure alcohol, hypertonic saline, and silver nitrate) have been used for inactivation of the hydatid cyst contents; nevertheless most of such compounds may produce a variety of side effects [3, 6, 7]. Hypertonic glucose is a cheap and available material, and due to the possibility of intravenous injection, it is with low complication and a trusted substance [8]. In addition, hypertonic glucose is reported to be a successful protoscolicidal agent [8–12]. So far, the protoscolicidal efficacy of hypertonic glucose at different concentrations and exposure times has not been tested by in vitro studies. Therefore, this study was undertaken to evaluate the protoscolicidal effects of hypertonic glucose on protoscolices of hydatid cyst at different concentrations and exposure times.

## 2. Materials and Methods

### 2.1. Collection of Protoscolices

The fluid of hydatid cysts was gathered aseptically from the livers of slaughtered sheep at Qazvin abattoir, in the central of Iran. Fertility of the cysts was assessed and protoscolices of hydatid cysts were washed three times in normal saline. Viability of protoscolices was confirmed prior to the experiments. The viability of protoscolices was determined by their motility characteristics and with 0.1% eosin staining under light microscopy. When the percentage of viable protoscolices was greater than 90%, they were considered to be appropriate for our study.

### 2.2. Scolicidal Assay

In this study, the protoscolices were exposed to different concentrations of hypertonic glucose (10%, 15%, 20%, 25%, 30%, 40%, and 50%) at room temperature for different times (1, 2, 3, 4, 5, 6, 7, 8, 9, 10, 20, 30, 40, 50, and 60 min). Briefly, in each experiment, 2.5 mL of each concentration of hypertonic glucose was placed in a test tube, to which a drop of protoscolex-rich sediment (containing at least 1000 protoscolices) was added using a Pasteur pipette. The content of each tube was gently mixed and incubated. At the end of each incubation time (1, 2, 3, 4, 5, 6, 7, 8, 9, 10, 20, 30, 40, 50, and 60 min), 10 mL of normal saline was added to each tube and centrifuged for 1 min at 300 rpm and the supernatant was discarded. The same procedure was repeated 6 times. One milliliter of 0.1% eosin stain was then added to the remaining settled protoscolices, mixed gently, incubated for 15 min, and followed by careful removal of the upper portion. Later, the remaining protoscolices pellet was smeared on a manually scaled glass slide, covered with a cover glass, and examined under a light microscope. The percentages of dead protoscolices were determined by counting an average of 1000 protoscolices. Hypertonic saline (20%) and normal saline were used as positive and negative controls, respectively. All experiments were performed six times [5, 13].

### 2.3. Statistical Analysis

Data are represented as mean ± standard deviation. Statistical analyses were performed by one-way analysis of variance (ANOVA) and *t*-test to express the difference among the groups. All analyses were performed using SPSS software version 16. Data were considered statistically significant at *P* < 0.05.

## 3. Results

The scolicidal effect of hypertonic glucose at different concentrations (10%, 15%, 20%, 25%, 30%, 40%, and 50%) and exposure times (1, 2, 3, 4, 5, 6, 7, 8, 9, 10, 20, 30, 40, 50, and 60 min) is shown in Table 1. Hypertonic saline (20%) and normal saline were used as positive and negative controls, respectively.

**Figure 1:**
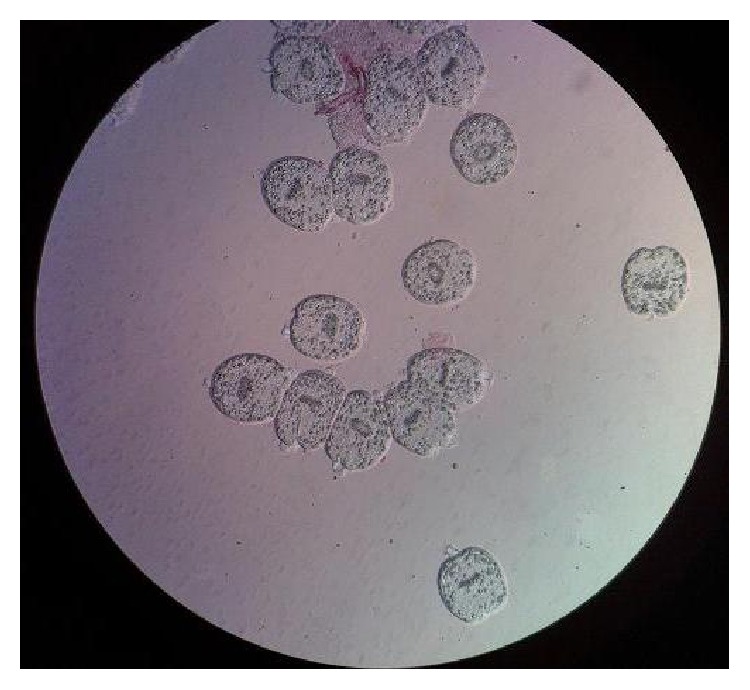
Live protoscolices after staining with 0.1% eosin.

**Figure 2:**
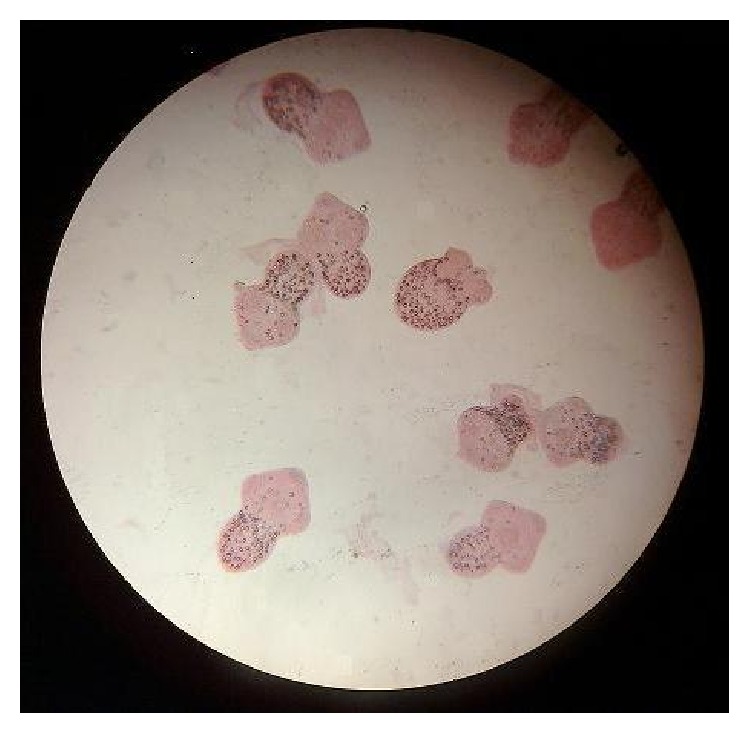
Dead protoscolices after 40 min exposure time with 40% glucose.

Glucose at 10%, 15%, 20%, 25%, and 30% concentrations had a little effect on protoscolices after 1, 2, 3, 4, 5, 6, 7, 8, 9, and 10 min exposure times. Concentrations of 10% and 15%, even after 60 min exposure time, failed to show a considerable effect on protoscolices, but glucose at 20% and 25% concentrations after 40 min exposure time killed more than 50% of protoscolices. In addition, the mortality rates of 30% glucose following 30, 40, 50, and 60 min exposure times were 52.1%, 61.7%, 64.1%, and 86%, respectively. All protoscolices were killed after 40 min exposure time with 40% glucose as shown in Figure 1 (live protoscolices) and Figure 2 (dead protoscolices). The scolicidal effect of such glucose concentration after 20 and 30 min was 98.8% and 99.6%, respectively. Glucose at 50% concentration, after 1–5 min exposure time, killed almost 50% of protoscolices, but after 6, 7, 8, 9, 10, and 20 min exposure times, the scolicidal effect of this glucose solution was 63.9%, 67.2%, 83.4%, 95.3%, 97.4%, and 100%, respectively. The results of this study showed that the scolicidal effect of all seven concentrations of glucose was significant comparing with the control groups at all exposure times (*P* < 0.001). Moreover, the protoscolicidal effects of different concentrations of glucose except for the 40% and 50% concentrations in various exposure times, compared with the effect of 20% hypertonic saline, were found to be significant (*P* < 0.001). Therefore glucose at concentrations of 40% and 50%, like hypertonic saline 20%, killed all (100%) protoscolices after 40 and 20 min exposure times, respectively.

## 4. Discussion

In this study, the protoscolicidal effect of glucose at various concentrations was assessed at different exposure times and the results were statistically found to be significant compared with the control groups. The fatality rate of glucose at different concentrations on protoscolices was further examined by increasing the exposure times which demonstrated the effect of time on eliminating the viability of protoscolices. Moreover, our results showed that, at a fixed exposure time, increasing the glucose concentrations led to reduced viability of protoscolices, a finding in agreement with the results of other researchers [5, 14–16]. Of course, glucose in some gradually increasing concentrations including 10%, 15%, 20%, 25%, and 30% and exposure times such as 1, 2, 3, 4, 5, 6, 7, 8, 9, and 10 min produced a little effect on protoscolices with no significant difference. Meanwhile, other studies also have shown that glucose at 10%, 15%, and 25% concentrations is without any obvious protoscolicidal effect after 1, 2, and 5 min exposure times [12]. Based on our results, glucose concentrations of 10% and 15%, even after 60 minutes of exposure time, had no significant scolicidal effect on the protoscolices, implying the absence of glucose effect on protoscolices at low concentrations. Glucose at 20% and 25% concentrations after 60 minutes killed more than 50% of protoscolices, indicating that higher glucose concentrations following longer exposure times were more effective compared to lower glucose concentrations. The viability of about 50% and 85% of protoscolices disappeared after 30 and 60 min exposure times with 30% glucose, respectively. In addition, our results showed that glucose at 30% concentration could significantly eliminate the viability of protoscolices after 60 min exposure time; therefore the possibility of higher elimination of protoscolices following longer exposure times remains as a subject which needs further investigations.

In the present study, glucose at 40% concentration had no significant effect on protoscolices at exposure times less than 20 minutes, but after 20, 30, and 40 min this concentration of glucose killed 98.8%, 99.6%, and 100% of protoscolices, respectively. So far, no study has been carried out regarding the effect of 40% glucose on protoscolices; therefore this concentration could be suggested as a suitable alternative protoscolicidal agent in future studies. Moreover, 50% glucose concentration in 1 to 5 min exposure times killed approximately 50% of protoscolices, and, in 6 to 8 min, this effect was ascending, but, in 9 to 10 min exposure times, a significant amount of protoscolices died out. Hosseini et al. indicated that the significant effect on protoscolices occurred in 1 to 5 minutes exposure time [12]. In our study, after a 20 min exposure time, all protoscolices lost their viability, but in other studies it was mentioned that such effect happened after 15 and 30 minutes [8, 9]. This difference in effectiveness could be related to diversity of parasite strains and differences in geographic location and experimental conditions; however, further studies are required.

Hypertonic glucose was initially used as a scolicidal agent on pericardial hydatid cyst of human by Ferrini et al. [10]. Moreover, Tejada et al. demonstrated that the use of hypertonic glucose produced no apparent symptoms [11]. In this regard, there are other studies in which 10% and 25% glucose solutions as well as normal saline were injected into the peritoneal cavity of rats and after 6, 24, and 48 hours, the peritoneal and mesenteric tissue of rats were examined for fibrosis and necrosis. Although glucose and normal saline solutions in the peritoneal cavity produced, to some extent, nonspecific inflammation but fibrosis or necrosis in the mesentery and parietal peritoneum was not seen and the degree of inflammation caused by 10% and 25% glucose and normal saline was equal [17]. In addition, Hosseini et al. in similar experiments following injection of 10%, 15%, 25%, and 50% glucose concentrations into the gallbladder of rabbits showed that hypertonic glucose is a harmless substance and does not lead to the fibrosis of bile ducts and hepatic necrosis [18]. Therefore, it is suggested that hypertonic glucose could be a suitable replacement for lots of protoscolicidal materials with side effects.

On the other hand, hypertonic saline at 20% concentration, which destroys the wall of hydatid cyst by severe osmotic properties, is widely used today as a scolicidal agent [19]. In this study, hypertonic saline, compared with different concentrations of glucose, had a significant effect on the protoscolices within the first minutes of exposure time. Hypertonic saline, in our study, killed 97% of protoscolices in 10 minutes, but the total destruction of protoscolices was obtained after 20 minutes; however, this time course varies in different studies. In some other studies, the killing of all protoscolices occurred after 2, 4, 15, and 45 min exposure times [8, 13, 14, 20]. Although 20% hypertonic saline is an effective protoscolicidal agent compared with the hypertonic glucose used in this study and other studies [13, 14, 20], nevertheless the dangerous side effects such as peritoneal inflammation, fibrosis of bile ducts, liver necrosis [14, 19], and the sharp rise in blood sodium have been reported after injection of hypertonic saline [21–23], and the use of alternative materials is required.

## 5. Conclusion

In the present study, although the effect of hypertonic saline on protoscolices was more than hypertonic glucose in lower exposure times, but the effect of both 20% hypertonic saline and 40% hypertonic glucose was 100% after 20 min exposure time. It seems that using hypertonic glucose, compared with hypertonic saline, can be preferred as a protoscolicidal agent. Therefore, our findings of in vitro study indicated that 40 and 50% hypertonic glucose could be an appropriate protoscolicidal agent for surgical operations; however to support in vitro findings, in vivo studies and further investigations are suggested.

## Figures and Tables

**Table 1:** Protoscolicidal effects of glucose at different concentrations and exposure times in comparison with 20% hypertonic saline and normal saline.

Agents	Number of tests	% (average of dead protoscolices ± SD)															
		Exposure times															*P* value
		1 min	2 min	3 min	4 min	5 min	6 min	7 min	8 min	9 min	10 min	20 min	30 min	40 min	50 min	60 min	
10% glucose	6	3.3 ± 1.4	4.3 ± 1.4	7.1 ± 0.34	7.3 ± 0.66	6.7 ± 0.6	8.4 ± 0.92	10.9 ± 0.9	9.3 ± 0.67	10.4 ± 0.75	10.5 ± 1.18	1.7 ± 0.83	12.8 ± 0.56	14.7 ± 0.52	14.8 ± 0.81	17 ± 0.97	*P* < .001
15% glucose	6	3.3 ± 0.23	5.2 ± 0.71	5.6 ± 0.76	6.8 ± 0.84	6.8 ± 0.83	10.8 ± 0.57	11.1 ± 0.69	12.2 ± 0.77	12.8 ± 0.77	13 ± 0.71	13 ± 0.71	16.9 ± 0.77	19.7 ± 0.66	25.3 ± 0.85	31.7 ± 0.78	*P* < .001
20% glucose	6	8.1 ± 0.68	11.8 ± 0.84	11.8 ± 1.3	13.6 ± 0.7	14.3 ± 1.2	14.1 ± 0.68	12.5 ± 0.7	14.8 ± 1.2	15.2 ± 0.71	16.1 ± 0.62	15.6 ± 0.86	24.7 ± 1.3	28.1 ± 1.3	36.6 ± 1.1	54.3 ± 0.67	*P* < .001
25% glucose	6	9.2 ± 0.81	11.2 ± 0.69	11.9 ± 0.66	12.8 ± 1.3	12.7 ± 0.77	14.4 ± 0.92	14.3 ± 1.9	15.1 ± 0.62	16.4 ± 0.69	16.5 ± 1	21.4 ± 0.47	32.5 ± 0.71	40.4 ± 0.89	44.2 ± 0.52	74.1 ± 1.5	*P* < .001
30% glucose	6	11.7 ± 0.57	12.8 ± 0.79	13.1 ± 0.7	14.4 ± 1.1	14.3 ± 0.73	16.8 ± 0.84	17.9 ± 1.52	17.2 ± 0.81	17.1 ± 0.7	17.7 ± 0.59	30.9 ± 1.5	52.1 ± 0.7	61.7 ± 0.57	64.1 ± 1.32	86 ± 0.71	*P* < .001
40% glucose	6	14.1 ± 1.44	14.3 ± 0.55	15.3 ± 0.83	15.3 ± 0.73	16.7 ± 1.37	20.2 ± 1.41	28.4 ± 1.91	30.4 ± 1.56	35.2 ± 0.77	40.7 ± 0.49	98.8 ± 0.38	99.6 ± 0.14	100 ± 0	100 ± 0	100 ± 0	*P* < .001
50% glucose	6	51.4 ± 1.56	52.6 ± 2.1	52.8 ± 1.84	53 ± 2.3	53.5 ± 2.42	67.2 ± 2.64	83.4 ± 0.7	95.3 ± 1.33	97.4 ± 0.68	100 ± 0	100 ± 0	100 ± 0	100 ± 0	100 ± 0	100 ± 0	*P* < .001
20% saline	6	69.8 ± 1.97	72.6 ± 1.66	75.6 ± 2.03	81.5 ± 1.61	85.9 ± 1.47	87.7 ± 0.83	88.2 ± 1.41	91.8 ± 1.27	94.6 ± 1.34	97 ± 0.7	100 ± 0	100 ± 0	100 ± 0	100 ± 0	100 ± 0	*P* < .001
Normal saline	6	2.1 ± 0.28	2 ± 0.22	1.9 ± 0.14	1.8 ± 0.14	1.7 ± 0.14	1.8 ± 0.28	2 ± 0.14	1.7 ± 0.22	2.1 ± 0.2	1.9 ± 0.14	1.8 ± 0.14	1.7 ± 0.15	1.9 ± 0.14	2 ± 0.14	2 ± 0.15	*P* < .001
